# A case of choledochal cyst type IV

**DOI:** 10.1016/j.radcr.2024.10.033

**Published:** 2024-11-07

**Authors:** Dian Komala Dewi, Oki Kurniawan, Dudus Indra Gunawan, Harry Galuh Nugraha

**Affiliations:** a Department of Radiology, Faculty of Medicine Padjadjaran University, Dr. Hasan Sadikin Hospital Bandung, West Java, Indonesia; b Department of Pediatric Surgery, Faculty of Medicine Padjadjaran University, Dr. Hasan Sadikin Hospital Bandung, West Java, Indonesia

**Keywords:** Coledocal cyst, Icteric, Children

## Abstract

A choledochal cyst (CC) or biliary cyst is a congenital or acquired anomaly affecting the biliary tree. It involves the dilation of the biliary tree that could affect the extrahepatic and/or the intrahepatic segments. A choledochal cyst (CC) has traditionally been considered as a cystic dilation of the extrahepatic bile duct. The incidence of choledochal cysts is high in the Asian population with a female predominance. Choledochal cysts can present at any age, including infancy. However, 80% of choledochal cysts are diagnosed in the first decade of life, with cholestasis being the most common sign in infants, and cholangitis or pancreatitis being less common. Radiological and endoscopic imaging is the cornerstone of CC diagnosis. We report a case of 16 years old patient with choledocal cyst. The case has distinct clinical signs that are easily recognizable.

## Introduction

Choledochal cysts are intrahepatic, extrahepatic or both biliary tree dilatations. The incidence in the western population is 1 in 100,000-150,000 live births; in Asian populations, 1 in 1,000-13000, Japan has two-thirds of all cases [1]. There is predominance in the female population, which is more common in the first decade [2]. In Indonesia, epidemiological data on choledochal cysts and biliary atresia are still rarely reported. However, a study conducted at Cipto Mangunkusumo Hospital showed that biliary atresia is the most common cause of obstructive cholestasis (>90%). According to the study, 60 patients with biliary atresia were treated at the Department of Pediatrics at Cipto Mangunkusumo Hospital over the past 12 years (from 1998 to 2009). Out of these patients, only 20% sought treatment before the age of 2 months [3]. Choledochal cysts (CCs) are typically diagnosed during childhood, with only 25% of cases found in adults, although this number has been rising in recent years. The classic triad of abdominal pain, a palpable abdominal mass, and jaundice is observed in only 20% of cases, with 85% of children exhibiting 2 of these symptoms, most commonly abdominal mass and jaundice, compared to just 25% of adults. Infants under 12 months often present with jaundice, acholic stools, and vomiting, while abdominal pain is the most common symptom in adults. No laboratory tests specifically diagnose CCs, but imaging plays a crucial role. Ultrasound, often used initially, is effective in detecting CCs but may miss certain conditions like anomalous pancreaticobiliary junctions (APBJ). CT cholangiography offers better accuracy for type IV and V cysts and aids in surgical planning. The Technetium-99 HIDA scan is particularly useful in distinguishing CCs from biliary atresia in neonates. MRCP is the gold standard for diagnosing CCs due to its high sensitivity and non-invasive nature, although it has limitations in detecting minor ductal abnormalities. ERCP, while highly accurate, is less commonly used due to its invasive nature and associated risks, but it remains valuable for both diagnosis and treatment, especially for type III CCs where endoscopic sphincterotomy can be performed [4].

## Case presentation

**Fig. 1:**
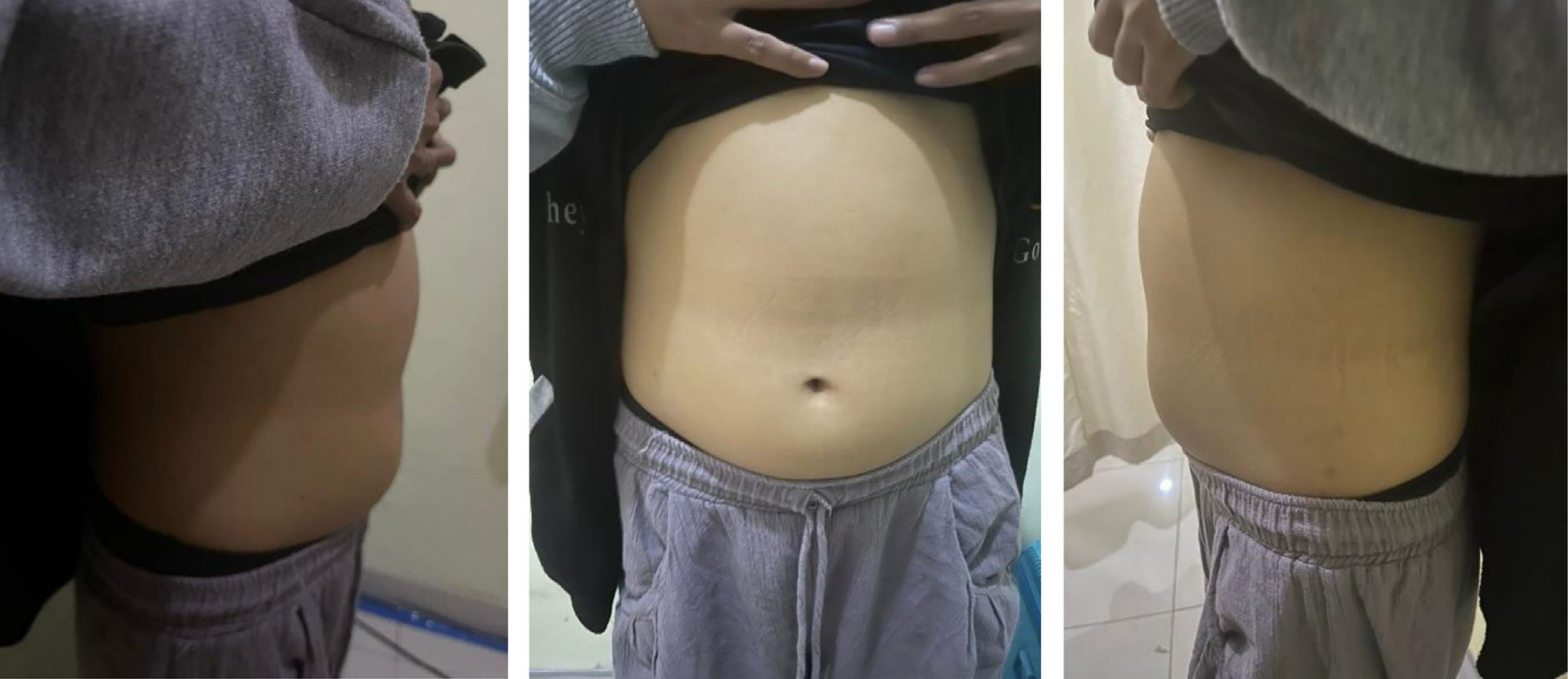
Clinical sign of patient of choledochal cyst type IV.

A 16-year-old girl complains of intermittent pain in the upper abdomen since last year. She also felt a lump in the upper left abdomen when pressed. Additionally, she had jaundice for the past year without fever. Her eyes is icteric. She feels bloated but does not experience stomach pain. The patient frequently feels nauseous and vomits, but can still pass gas. There are no abnormal bowel movement complaints, such as pale stools, difficulty defecating, diarrhea, pain during defecation, or bloody or mucous stools. She does not follow a high-fat or low-fiber diet. There are also urinary complaints, with urine the color of tea, but without pain during urination, difficulty starting urination, gritty urine, or blood in the urine. She does not feel incomplete after defecation. Her weight has not decreased drastically but also has not increased. There are no lumps elsewhere. (Fig. 1) The patient has no history of tattoos or blood transfusions.

On an MSCT scan of the abdomen and pelvis, there is evidence of intrahepatic and extrahepatic biliary ectasia, accompanied by multiple cystic lesions in the right intrahepatic duct and the common bile duct. Hepatosplenomegaly is present, along with mild dilation of the splenic vein. There are multiple enlarged lymph nodes in the right abdominal mesentery. The pancreas, kidneys (bilateral), urinary bladder, and rectum show no abnormalities. Subsequently, a hepatobiliary ultrasound was performed, revealing cystic lesions apparently connected to the extrahepatic bile duct, along with intrahepatic biliary ectasia, suggesting a suspected choledochal cyst type IV. Splenomegaly was also noted, while the liver, pancreas, and gallbladder appeared normal on the ultrasound. In addition, the patient underwent laboratory tests. There was an increase in SGOT, SGPT, total bilirubin, direct bilirubin, and indirect bilirubin. There was a decrease in urea. The patient's SGOT reached 133 U/L, SGPT 70 U/L, total bilirubin 9.458 mg/dl, direct bilirubin 6.635 mg/dl (n=0.1-0.3 mg/dl), and indirect bilirubin 2.823 mg/dl (n=0.2-0.8 mg/dl), while the urea level was low (16.4 mg/dl).

**Fig. 3:**
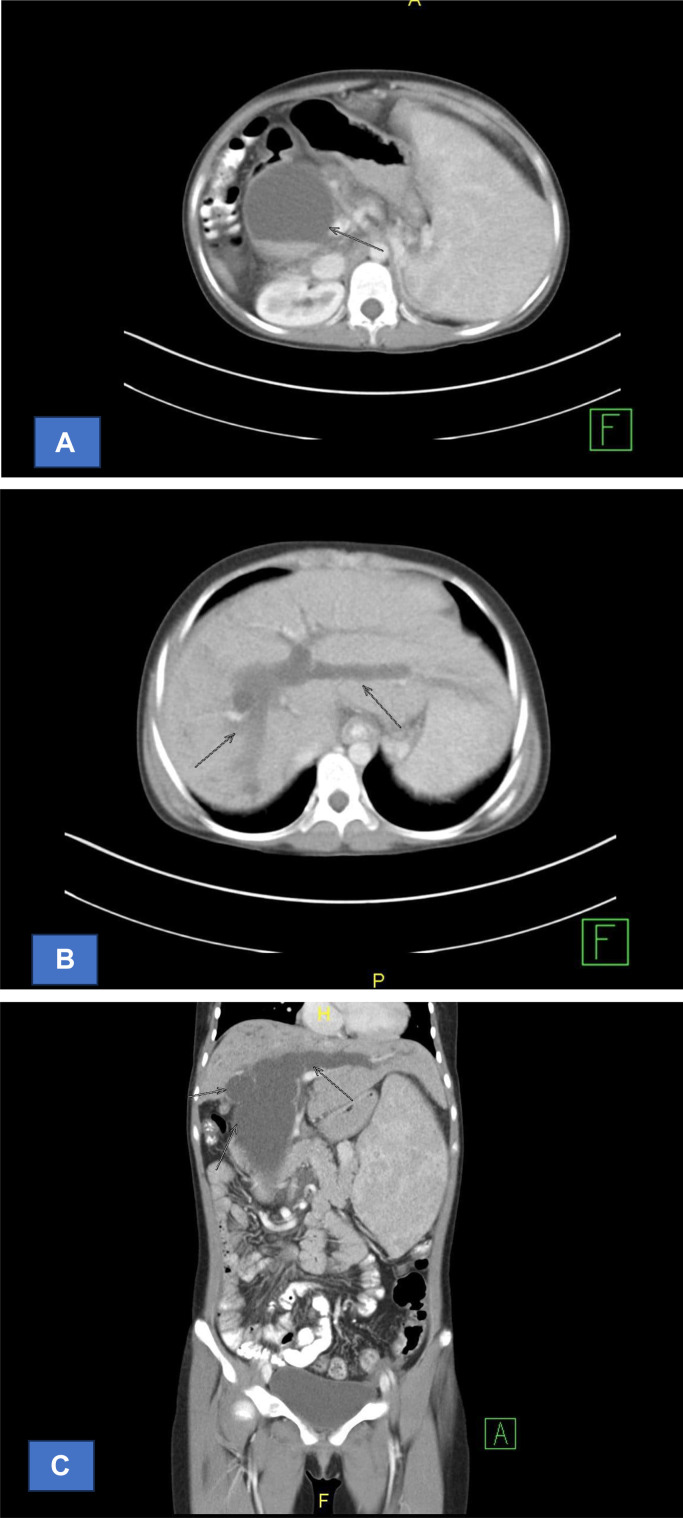
CT scan: sagital intra and extra hepatal duct, patients with Choledochal Cyst type IV: (A) The axial cut shows the dilation of the intra and extrahepatal ductus, (B) The axial cut shows the dilation of the intahepatal ductus, (C) The coronal cut shows the dilation of the intahepatal ductus, size of the lesion has not increased, the wall is not thickened, and the edges are regular. No masses or stones are visible. The extrahepatic bile duct is dilated, with a cystic lesion containing bile sludge, measuring approximately 6.25 × 7.14 × 12.40 cm, located in the proximal to distal common bile duct.

An MSCT scan of the abdomen and pelvis with axial slices of 5 mm thickness, reconstructed into coronal and sagittal views also of 5 mm thickness, was performed using 500 ml of oral contrast and 50 ml of IV contrast. The scan revealed intrahepatic and extrahepatic biliary ectasia with multiple cystic lesions in the right intrahepatic duct and the common bile duct, indicative of Choledochal Cysts Type IV. Hepatosplenomegaly was present with mild dilation of the splenic vein. Multiple enlarged lymph nodes were observed in the right abdominal mesentery. No abnormalities were found in the pancreas, bilateral kidneys, urinary bladder, and rectum (Fig. 3).

**Fig. 4:**
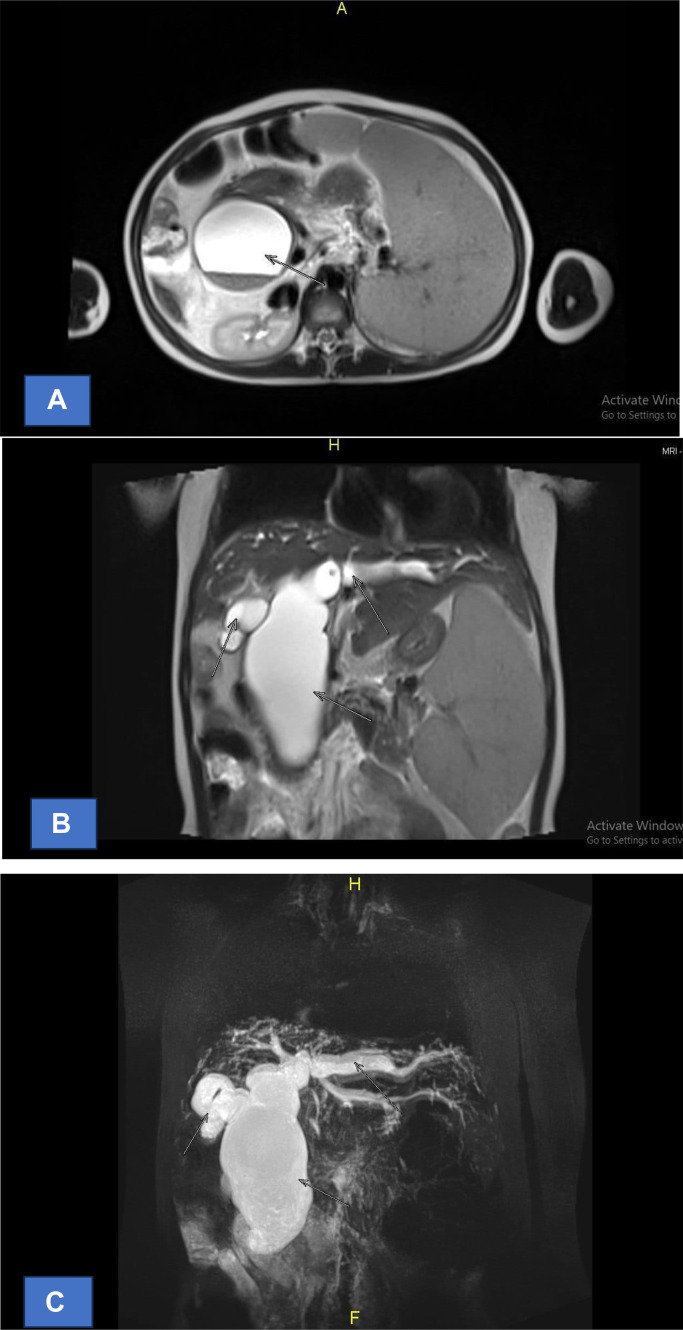
MRCP: intra and extrahepatal duct, patients with Choledochal Cyst type IV: (A) The axial cut shows the dilation of the extrahepatal ductus, (A) The axial cut shows the dilation of the extrahepatal ductus. (B) The biliary tree cut shows the dilation of the intahepatal ductus, (C) The coronal cut shows the dilation of the intahepatal ductus.

Three months later, an MRCP was conducted, showing multiple cystic lesions forming a fusiform appearance in the bilateral intrahepatic bile ducts and extrahepatic bile ducts, indicating a choledochal cyst Type IVA according to the Todani classification. Splenomegaly was noted, while the liver and pancreatic ducts were within normal limits (Fig. 4).

**Fig. 2:**
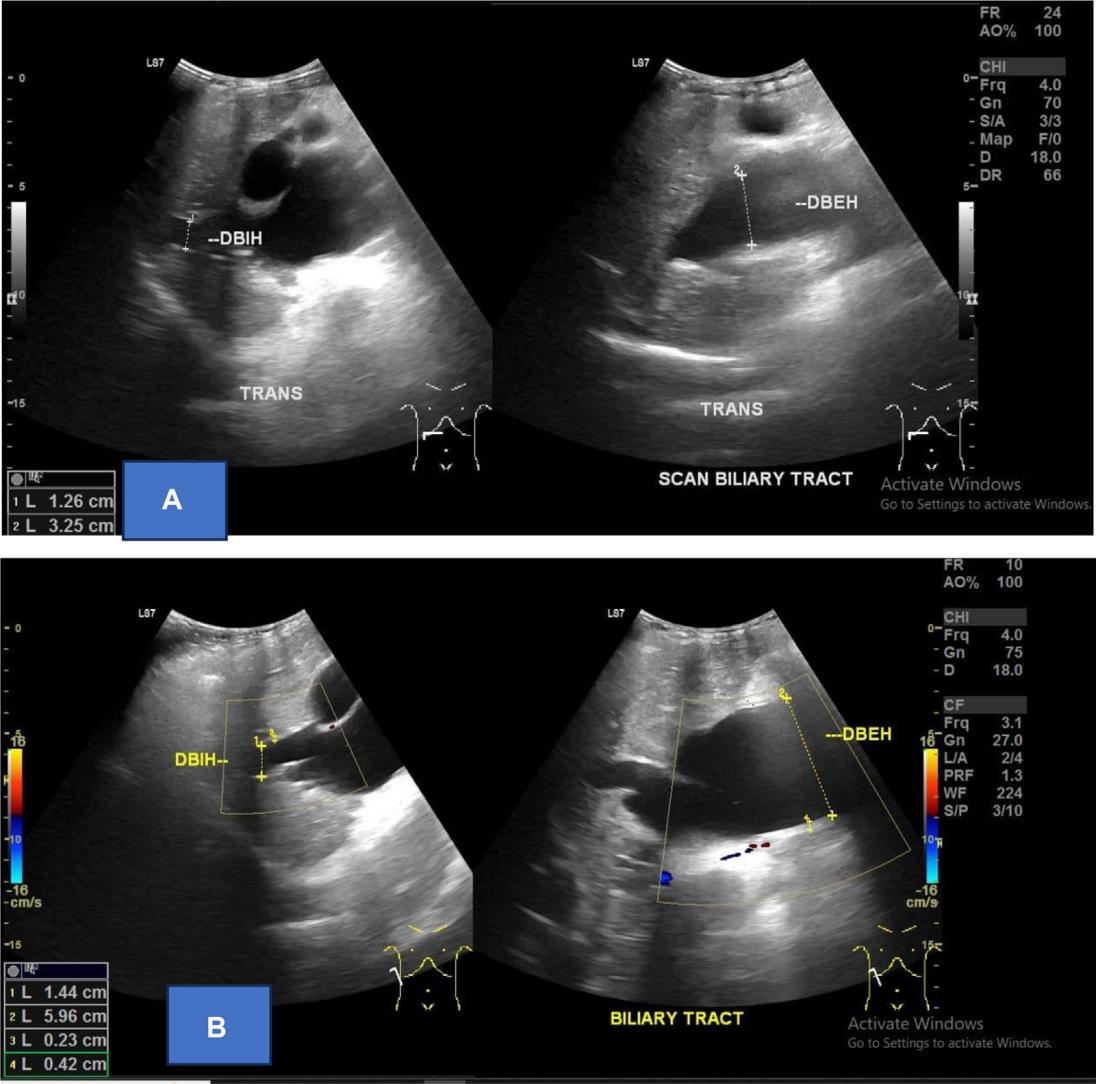
Intrahepatal and extrahepatal ductus US, patients with Choledochal Cyst type IV (A) Ultrasound showed dilatation of intrahepatal and extrahepatal ductus, (B) doppler showed without vascularization.

Ultrasound of abdomen shows The lesion appears as an anechoic area with well-defined borders, regular edges, measuring approximately 9.46×4.73 cm, and seems to be associated with the extrahepatic bile duct. No vascularization is observed on color Doppler examination. The intrahepatic bile ducts are dilated (Fig. 2).

A fusiform cystic lesion is observed in the common hepatic duct to the common bile duct, with a maximum diameter of approximately 6.34 cm. The lesion shows hypointense signal changes on T1-weighted images (T1W1) and hyperintense signal changes on T2-weighted images (T2W1). Additionally, multiple fusiform cystic lesions are present in the right and left intrahepatic ducts, with the largest diameter being about 1.94 cm. These lesions also exhibit hypointense signals on T1W1 and hyperintense signals on T2W1.

## Discussion

The term choledochal cyst refers to a spectrum of congenital biliary tract disorders that were previously grouped under the name idiopathic dilatation of the common bile duct. Adults with initial manifestation of choledocal cysts usually have nonspecific right upper quadrant symptoms, jaundice, pancreatitis or cholangitis. A palpapble mass, which is a common presentation in children is rare. Diagnosis can be made by USG, CT-scan, ERCP dan MRCP. Ultrasonography was performed as initial screening method, CT-scan to establish the diagnosis. The treatment of a choledochal cyst has changed. In the past, a cysto-jejunostomy was the standard procedure [5]. Diagnosis of choledochal cyst suggesting biliary or pancreatic origin including right upper quadrant pain, jaundice, pancreatitis and nausea and vomiting are also common. There are classical symptoms of children with choledochal cyst, it is abdominal pain, jaundice or an abdominal mass. A recent series evaluating choledochal cysts in pediatric and adult patients identified abdominal pain as the presenting complaint in 61% of patients, which was the most common symptom in both adults (72%) and children (41%) [6]. In this case, patient complains there in abdominal pain and feel a lump in her abdomen at the last past year. Patient has jaundice and her eyes icteric. the patient frequently feels nauseous and vomit. In a multi-institutional analysis of 394 patients who underwent resection of CCs, 84.5% of patients with CC presented with symptoms while 15.5% of individuals were asymptomatic. Adults were more likely to present with abdominal pain, while children were more likely to present with jaundice. Other CC-related symptoms may include cystolithiasis, cholecystitis, pancreatitis, liver abscess, and liver cirrhosis [1].

There are no laboratory studies are specific for choledochal syst [4]. A choledochal cyst is a bile duct anomaly that disrupts the transportation of bile from the liver to the gallbladder and small intestine. Billiary obstruction refers to obstruction n the biliary system, leading to blockage of bile flow into the small intestine. It can occur anywhere along the path from the liver to intestinal tract. Biliary obstruction is common and affect a large portion of the population around the world. The manifest of this process is causing jaundice. Jaundice, a physical exam finding of yellowish discoloration of the skin, conjunctive, or mucous membranes is a consequence of obstruction leading to bile stasis and buildup of conjugated bilirubin in the blood. Clinically, jaundice is evident of total serum bilirubin values at 3 mg/dl [7]. In this case, total bilirubin of patient is 9.458 mg/dl.

Radiological examinations are essential for confirming the diagnosis and ruling out differential diagnoses, such as biliary duct obstruction, potential biliary malignancy, or metastasis. The gold standard for diagnosing choledochal cysts (CC) is Magnetic Resonance (MR) and Magnetic Resonance Cholangiopancreatography (MRCP), which allow for detailed visualization of intrahepatic and extrahepatic structures, the pancreaticobiliary junction, and potential complications arising from CC. However, the availability of MR in Indonesia is currently limited to major cities. In contrast, ultrasound (USG) is more widely accessible, cost-effective, noninvasive, and can be used as an initial modality to evaluate the biliary ducts, with a sensitivity of 70-97% for detecting the classic triad and assessing for complications [8].

The Todani classification is a widely recognized system for categorizing choledochal cysts, identifying 5 main types with several subtypes, some of which may not be pathologically related. Type I, the most common, accounts for 80-90% of cases and involves dilatation of the extrahepatic bile duct, sometimes detectable in utero. This type is further divided into 3 subtypes: Ia (dilatation of the entire extrahepatic bile duct), Ib (dilatation of a specific segment), and Ic (dilatation of the common bile duct portion). Type II is marked by a true diverticulum arising from the extrahepatic bile duct, while Type III, or choledochocele, involves dilatation within the duodenal wall. Type IV is the next most prevalent and is subdivided into IVa, involving cysts in both intra and extrahepatic ducts, and IVb, which features multiple dilatations of the extrahepatic ducts alone. Type V, known as Caroli disease, includes multiple dilatations in the intrahepatic ducts, and Type VI refers to the dilatation of the cystic duct [9]. Although the Todani classification is commonly used, it has been criticized in surgical literature for possibly grouping distinct conditions into an overly simplified grading system. The Komi classification offers an alternative by categorizing choledochal cysts based on the anomalous union of the pancreatic-bile duct (AUPBD) into 3 types [10]. In this case, the patient was diagnosed with a type IV choledochal cyst during an ultrasound examination, as it involved both the extrahepatic bile duct and intrahepatic biliary ectasia. This was also demonstrated in the MRCP examination, which revealed multiple cystic lesions forming in the bilateral intrahepatic bile ducts and extrahepatic bile ducts, indicating a type IVA choledochal cyst according to the Todani classification.

In conclusion, choledochal cysts (CCs) are biliary tract disorders more common in Asian populations, often diagnosed in childhood. They present with symptoms like abdominal pain, jaundice, and masses, varying by age. Imaging, especially MRCP, is key to diagnosis, with the Todani classification widely used despite some criticisms. In this case, a type IVA choledochal cyst was identified through ultrasound and confirmed by MRCP. Early diagnosis is crucial for effective management, highlighting the need for accessible imaging technologies, particularly in regions with limited resources.

## Patient consent

Written informed consent for publication of their case was obtained from our patient's parent.

## References

[1] Brown ZJ, Baghdadi A, Kamel I, Labiner HE, Hewitt DB, Pawlik TM (2023). Diagnosis and management of choledochal cysts. HPB (Oxford).

[2] Tariq WB, Twayana AR, Sunuwar N, Anjum A, Deo S, Rayamajhi S (2022). Case report: a rare case of choledochal cyst. F1000Res.

[3] Juju J, Efa A, Eka W, Fauziah R (2022).

[4] Hoilat GJ, John S (2024). StatPearls.

[5] Al-Ba'adani MN, Alammari SA, Jumman NA (2022). Choledochal cysts diagnosis and treatments: case report. OAlib.

[6] Ronnekleiv-K SM, Soares KC, Ejaz A, Pawlik TM (2016). Management of choledochal cysts. Curr Opin Gastroenterol.

[7] Priska VK, Indira Prawita M (2022). Choledocal cyst pada dewasa: peran ultrasonografi. Cendana Med J.

[8] Sharma R, Gaillard F. Choledochal cyst. In: Radiopaedia.org; 2008.

[9] Mohammad NK Disorders of the liver (2024). Avery’s Diseases of the Newborn Christine Gleason, M.D. is an Academic Neonatologist and Professor Emeritus of Pediatrics at University of Washington and Seattle Children’s Hospital.

[10] Coucke EM, Akbar H, Kahloon A, Hina Akbar M (2022). In: StatPearls [Internet]. Treasure Island (FL): StatPearls Publishing; 2024 Jan.

